# Overt Oculomotor Behavior Reveals Covert Temporal Predictions

**DOI:** 10.3389/fnhum.2022.758138

**Published:** 2022-02-11

**Authors:** Alessandro Tavano, Sonja A. Kotz

**Affiliations:** ^1^Department of Neuroscience, Max Planck Institute for Empirical Aesthetics, Frankfurt am Main, Germany; ^2^Department of Neuropsychology and Psychopharmacology, Faculty of Psychology and Neuroscience, Maastricht University, Maastricht, Netherlands; ^3^Department of Neuropsychology, Max Planck Institute for Human Cognitive and Brain Sciences, Leipzig, Germany

**Keywords:** predictions, temporal expectations, eye movements, Parkinson's disease, hazard rate

## Abstract

Our eyes move in response to stimulus statistics, reacting to surprising events, and adapting to predictable ones. Cortical and subcortical pathways contribute to generating context-specific eye-movement dynamics, and oculomotor dysfunction is recognized as one the early clinical markers of Parkinson's disease (PD). We asked if covert computations of environmental statistics generating temporal expectations for a potential target are registered by eye movements, and if so, assuming that temporal expectations rely on motor system efficiency, whether they are impaired in PD. We used a repeating tone sequence, which generates a hazard rate distribution of target probability, and analyzed the distribution of blinks when participants were waiting for the target, but the target did not appear. Results show that, although PD participants tend to produce fewer and less temporally organized blink events relative to healthy controls, in both groups blinks became more suppressed with increasing target probability, leading to a hazard rate of oculomotor inhibition effects. The covert generation of temporal predictions may reflect a key feature of cognitive resilience in Parkinson's Disease.

## Introduction

Blinks are defined as the temporary closure (≈0.3 s) of both eyes via rapid movements of both the upper and lower lids: the closing and closed phases of the movement are extremely rapid (<0.1 s), while the opening phase is slower (≈0.2 s, Kwon et al., 2013). On average, healthy human adults blink every 3–5 s (≈12–20 blinks per minute, Fatt and Weissman, 1992). Blinks help preserve the integrity of the ocular surface (lubrication, shielding from light and dirt, relieving eye muscle fatigue, Hall, 1945). However, blink frequency far exceeds such basic physiological needs, and there is evidence that arousal and attention drive blink frequency to change depending on whether at any given moment sensory information processing can be chunked (Wascher et al., 2015), when a release of attention from external stimulation is required (Nakano et al., 2013), or when fulfilled expectations indicate the end of cognitive processing (Ichikawa and Ohira, 2004).

In general, spontaneous blink rates decrease when attention is directed to incoming, external stimuli, particularly during experimental trials (Van Opstal et al., 2016) and when sustained, continuous attention is required to successfully complete a task (Maffei and Angrilli, 2018). It is unclear whether the component of attention that modulates blinking probability is strictly under dopaminergic control (Maffei and Angrilli, 2018; Sescousse et al., 2018), but there is evidence that Parkinson's patients with dyskinetic symptoms often exhibit increased eye movement rates (Karson, 1983), possibly as a consequence of intracortical dishibition (Stinear and Byblow, 2003; Ammann et al., 2020). Indeed, eye movement disorders may present one of the early symptoms of Parkinson's Disease onset (Jung and Kim, 2019). However, while the blinking rate is likely a confounded measure as it could be due to either attentional demands or fatigue (Maffei and Angrilli, 2018), the temporal distribution of blink movements or blink timing appears to be a reliable and unconfounded index of participants' engagement in a task (Ichikawa and Ohira, 2004; Nakano and Miyazaki, 2019).

Temporal attention is reflected in the hazard rate distribution, which normalizes true stimulus probability using the survival function, that is, the probability that the event has not yet occurred (Luce, 1986). Recent work showed that blinks and saccadic movements are suppressed (oculomotor inhibition) before the onset of predictable targets (Abeles et al., 2020). For uniformly distributed stimulus onset times, the perceived probability of target onset is assumed to monotonically increase as time elapses. It follows that blink probability at each target position should diminish with increasing temporal expectations. As cortical beta disorganization in Parkinson's disease has been associated with reduced sensitivity to temporal regularities (te Woerd et al., 2015), and the generation of temporal expectations has been linked to motor cortical activity (Morillon and Baillet, 2017), we tested the distribution of blinks in Parkinson's patients (PD) and a healthy control group (HC) matched for gender, age, and cognitive performance. All participants completed an auditory task which required detecting the onset of a target sound in a continuous attention mode. Auditory stimulation sequences were composed of the continuous repetition of four standard tones followed by a fifth non-target, deviant tone. All sounds were delivered using a fixed stimulus onset asynchrony interval (isochronous stimulation). Target sounds occurred rarely (20% of sequences) and unpredictably (randomized distribution) within the repeating sequence, equiprobably substituting a standard tone in either position 2, 3, or 4, hence giving rise to the hazard rate of response times (see stimulus structure section). We hypothesized that if the orienting of attention in time giving rise to expectations depends on the functional integrity of motor cortical, then the distribution of blinks in time in PD and HC should differentially reflect the temporal statistics of target onset. Specifically, we expected PD patients to be less efficient than HC in suppressing blinks with the increasing probability of target onset as attention moved from position 2 to position 4 within each sound sequence.

## Materials and Methods

###  Participants

The experiment was conducted at the Max Planck Institute for Human and Cognitive Brain Sciences in Leipzig (Germany). Sixteen participants diagnosed with Parkinson's Disease (PD, 9 males, 7 females) were selected (mean age = 63.9 years, SD = 6.8). Sixteen healthy adult individuals (Healthy controls, HC), matched in age (mean = 63.9 years, SD = 7.1) and gender, were also recruited from the Institute's database. Education level was also matched (PD, mean = 5.6 years, SD = 1.2; HC, mean = 5.7 years, SD = 1.3). HC participants self-reported no neurological or psychiatric disorders or therapies involving the central nervous system. All participants signed a written informed consent complying with the Declaration of Helsinki on human experimentation. The study was approved by the Ethics Committee of the University of Leipzig, Germany.

###  Neuropsychological Profile

The two experimental groups were also cognitively matched on a battery of neuropsychological tests (see Table 1, reporting means and standard deviations within parenthesis): Mini Mental Test (Tombaugh and McIntyre, 1992); Tower of London (Shallice, 1982); Trail Making Test A and B (Tombaugh, 2004); Working memory–Digit Span Forward, maximal N of numbers recalled [Wechsler, 1997, Backward, maximal N of numbers recalled (Wechsler, 1997). For all pairwise comparisons, all ts_(30)_ ≤ -0.73, all ps ≥ 0.465].

**Table 1:** Neuropsychological results.

**Group**	**Mini Mental State**	**Tower of London**	**Trail Making Test A and B**	**Digit Span Fw**	**Digit Span Bw**
HC	29.12 (0.85)	16.18 (1.42)	A: 40.56 (12.06), B: 77.62 (25.32)	6.50 (1.00)	5.06 (0.89)
PD	28.87 (0.99)	15.43 (2.06)	A: 39.37 (12.21), B: 82.37 (32.49)	6.68 (1.04)	5.06 (1.39)

###  Clinical Profile

The average illness duration in participants with PD was 3.78 years (SD = 2.63), with only two participants having been diagnosed for more than 6 years (15 and 11 years). Most patients (11 out of 16) presented with both tremors and akinetic rigidity, while 3 presented solely with akinesia and 2 with tremors. The average Höhn and Yahr index (Höhn and Yahr, 1967) was 2.03 (SD = 0.53, range 1–3), suggestive of bilateral involvement preserved balance functions. Asymmetry in body symptoms was equally distributed (right side = 8). On the UPDRS motor scale (Goetz et al., 2007), the mean was 13.5 (SD = 5, range 7–21), indicative of minimal to mild slowness and movement abnormality. All participants with PD were pharmacologically treated, predominantly with Levodopa and Ergot-dopamine agonists.

###  Stimulus Structure

**Figure 1:**
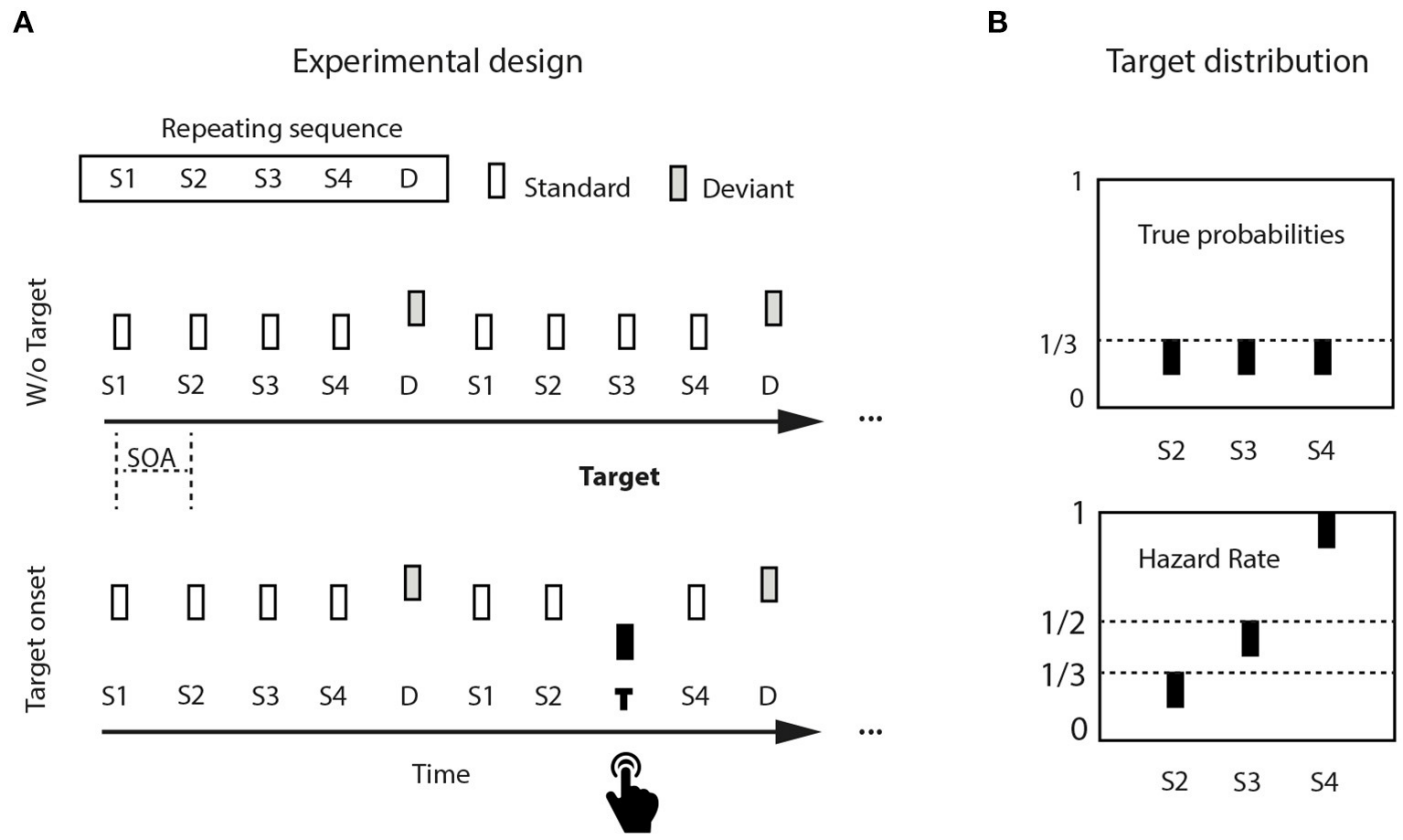
Experimental design: **(A)** Pure tones were isochronously distributed in continuously repeating five-tone sequences, composed of four standard tones (440 Hz) and a final non-target deviant tone (494 Hz). Target tones were lower in pitch (349 Hz), and appeared in 20% of the sequences (= 5% of the tones). **(B)** Tones would appear equiprobably either in position two, three, or four within a sequence (upper left panel). Potential rare target position is used as a proxy for elapsed time. The hazard rate distribution increases target probability with target position (upper right panel).

Stimuli were three 50-ms pure tones (5 ms rise/fall), organized into continuously repeating five-tone sequences, binaurally presented via loudspeakers at 80 dB SPL and generated using Matlab (version 7, Mathworks, Natick, MA). The five-tone sequence was composed of four standard tones followed by a non-target deviant tone (Figure 1A). Standards were 440 Hz in pitch (A4 on the equal tempered scale, presented 900 times, 75% global stimulus probability), non-target deviants were 494 Hz (B4, presented 240 times, 20% global stimulus probability). A rare target (349 Hz, F4, presented 60 times, 20% of sequences, 5% global stimulus probability) occurred equiprobably (1/3) at one among standard positions 2, 3, or 4. To detect a target, participants had to attentively listen to each incoming sequence, whether it contained a target or not; within each sequence, internal target probability was predicted to changed with elapsed time, generating a hazard rate distribution (Figure 1B, upper panel). Denoting the survival probability (“the event has not yet occurred”) as 1-F(t), where F(t) is the cumulative distribution function, the hazard function is then: h(t) = f(t)/(1-F(t)). There was a maximum of one target per sequence, and minimally two successive sequences without targets before the next target-containing sequence. Stimulus sequences were delivered using Presentation© software (version 12.0, www.neurobs.com) running on a Windows PC.

###  Experimental Design

Participants sat in an electrically shielded, sound-attenuated chamber, and fixated a white cross on a black computer screen at a distance of 1 meter while listening to the auditory sequences. They responded to target tone onset by pressing a button on an external response box, using their preferred hand. Participants were unaware of target distribution, and were instructed to respond to the onset of target tones as accurately and fast as possible by pressing a button on a response box. They trained in a short block of 60 experimental randomly distributed tone sequences containing three targets. The training phase was repeated maximally once. If errors were made (Missing, False Alarm), the training block was repeated until no errors were detected. Experimental tone sequences were delivered with a constant 750-ms stimulus onset asynchrony (SOA), corresponding to a 1.34 Hz stimulus rate (three 5-min blocks).

###  EEG Recording

Electroencephalographic (EEG) data were collected using a 26 scalp Ag/AgCl electrode set (BrainAmp, 10–20 system): Fp1, Fpz, Fp2, F7, F3, Fz, F4, F8, FT7, FC3, FC4, FT8, T7, C3, Cz, C4, T8, CP5, CP6, P7, P3, Pz, P4, P8, O1, O2. Two external electrodes were placed at right and left mastoid sites, and four additional electrodes were placed at both eye canthi (leftLateral, rightLateral), and above and below the right eye (lowerVertical, upperVertical) to record eye movements (electrooculogram, EOG). An online reference was placed on the left mastoid and the sternum served as ground. Electrode impedance was kept below 5 KOhm. EEG/EOG sampling rate was set to 500 Hz, with online high-pass filtering at 0.01 Hz. The resulting continuous recordings were visually inspected and pruned from non-stereotypical artifacts or extreme voltage changes values. An Independent Component Analysis (ICA, Infomax algorithm, Bell and Sejnowski, 1995, as implemented in the EEGLAB toolbox, Delorme and Makeig, 2004) was performed on pruned, offline highpass-filtered at 1 Hz and lowpass-filtered at 45 Hz (Kaiser window, Beta 5.6533, filter order points 9056 and 184, transition bandwidth 0.2 and 10 Hz, respectively), standardized (z-score) continuous data. Using the SASICA toolbox for EEGLAB (Chaumon et al., 2015), ICs reflecting blinks/vertical eye movements and lateral eye movements were identified by a correlation threshold of 0.7 with bipolarized vertical and lateral EOG channels. The SASICA toolbox also identified ICs likely to reflect muscle artifacts, using autocorrelation (lag = 20 s), as well as those reflecting bad electrodes via a measure of focal topography (threshold at 7 standard deviations relative to the mean across electrodes). The ICA results were then copied back to the pruned, standardized original continuous EEG data highpass-filtered at 0.1 Hz (lowpass 45 Hz). Eye-movement-related ICs, both vertical/blink-related and horizontal, ranged between 2 and 5 per participant, with at least a vertical/blink-related component per participant.

###  Blink Modeling

Blinks were individually modeled using the best signal selected out of a subset comprising the vertical EOG channel (both lowerV and upperV), a subset of frontal electrodes (in our case: Fp1, Fp2, Fz, F3, F4) and frontally focused independent components (ICs) representing blinks or vertical eye movements according to the Blinker toolbox pipeline (https://github.com/VisLab/EEG-Blinks; Kleifges et al., 2017). The Blinker algorithm first bandpasses the signal (1–20 Hz), then determines the intervals with an SD > 1.5 standard deviations above the signal mean (min interval = 50 ms, min separation between intervals = 50 ms). A fitting process follows by first finding specific landmarks for each blink interval, such as the maximal value within the interval, and the zero crossings immediately to the left and right of each max value, and then computing for each potential blink the best linear fits for the inner 80% of the up-stroke and down-stroke, respectively. The R^2^ of left and right fit lines with the actual blink trajectory measures how close the potential blink is to a stereotypical blink. Then, the blink-amplitude ratio (BAR) is computed by dividing the average amplitude of the signal between the blink left and right zero crossings by the average amplitude of the positive portion of the signal comprised between the preceding blink right zero crossing and the current blink left zero crossing, as well as the current blink right zero crossing and the following potential blink right zero crossing (or end of signal if the current blink is the last one). Potential blinks with a BAR outside the range [3–20] are not included in the final computation (“used” signal, see below).

Next, Blinker determines “good” blinks (upStroke and downStroke R2 > 0.90), “better” blinks (upStroke and downStroke R2 > 0.95), and “best” blinks (up-stroke and down-stroke R2 > 0.98). To eliminate extraneous eye movements from actual blinks, two further criteria are satisfied: 1) The positive amplitude by velocity ratio (pAVR = 3), calculated from the left zero crossing to the maximal amplitude of each blink, distinguishes between the sharp rise of saccades (large velocity) and the more curved one proper to blinks; 2) The maximum amplitude distribution criterion eliminates blinks with low R^2^ and with amplitude vastly away from the “best” blink median (Threshold = 5 robust standard deviations—1.48 times the median absolute deviation from the median—for “best” blinks, 2 for for “good” blinks). The resulting blinks constitute the “used” blinks set, which inform the analysis at an individual participant level (minimum number of blinks to stable estimates = 20).

###  Analysis of Blink Distributions

The Blinker pipeline was run on continuous, clean EEG datasets. One participant from the PD group was marked as an outlier as far as blink counts were concerned (*N* = 556) and thus was removed from further analysis, together which the gender- and age-matched HC participant. The final group was thus composed of 30 participants, 15 per group. Then, blink landmarks were copied back to the EEG trial structure, and finally epochs were extracted based on the repeating 5-tone sequences which did not contain a target (0–3,500 ms). This approach allowed analyzing the distribution of blinks in time as participants waited for a potential target, without any confounding effect from target onset. For each epoch, we marked the positions in time of blink maximal values (peaks), while the rest of the EEG data were zeroed out, obtaining vectors of blink peak distributions in time.

Participants were first compared for the total number of blinks (counts) and median blink-to-blink interval using a one-sided Wilcoxon rank sum test for equal medians, with the assumption that HC would outperform PD participants. The choice of a non-parametric statistical test was motivated by the non-gaussian distribution of blink counts (Kolmogorov-Smirnov test, all ps <1.645*10^-15^). Blink counts were subject to a robust regression analysis with bisquare weighting of the residuals (Matlab function *robustfit.m*), to asses the the relationship between HC and PD blink generators. The effect of age in driving blink counts was also tested, using both robust regression and Spearman correlation.

To assess the degree to which blink timing was sensitive to the auditory stimulus rate (1.34 Hz), blink epochs were concatenated into a single vector for each participant. A Fast Fourier Transform (FFT) analysis (*N* = 8192 data points, normalized dividing by N) was run on a hundred concatenated blink vector per participant in each group. The average peak power differences between HC and PD at sequence rate (0.267 Hz), stimulus rate (1.34 Hz), and first harmonic of the stimulus rate (2.67 Hz) were compared to their group threshold using a Wilcoxon signed rank test, and to each other using a one-sided Wilcoxon rank sum test (effect size *r* = Z/$\sqrt{Samplesize}$ for one sample/paired samples, *r* = Z/$\sqrt{Samplesize1+Samplesize2}$ for independent samples).

Next, we turned to the analysis of median blink distributions within the repeating sequence. First, for each participant blink peak latencies were binned using a 20-ms bin size. Then, bin counts were normalized by the total number of blinks, and smoothed using a moving median of 5 bins. To obtain a measure of regularity in blink distribution across the repeating sequence, we employed the Nelder-Mead simplex direct search algorithm (Image Analyst, 2021) and optimized the search for the best fit for 5 Gaussian distributions on the median distributions across participants in each group, using a sigma of 20 and taking each tone interval's middle point as an initial guess for the mean or peak of each Gaussian. We then calculated the dissimilarity between HC and PD median histograms using χ^2^ are a measure of distance: sum((xi-yi)^2^/(xi+yi))/2. We tested the significance of the distance value using a bootstrapping approach (1,000 randomizations). Then, for each participant we collected the value at the each grand median fitted Gaussian peak within each sound interval, and compared them across groups using a series of Wilcoxon rank sum tests, FDR-corrected. Finally, by regressing blink frequency against the positional order of potential Target stimulus onset (positions 2, 3, and 4), we obtained peri-stimulus estimates of hazard rate effects in blink distributions—from **–300 to** **+** **300 ms** relative to potential target onset -, which were tested for significance using a one-sample permutation test based on the t-statistic (Groppe et al., 2011; one-sided). Significance was determined for *p* = 0.05.

###  Probabilistic Saccade Estimation

As a partially independent measures of ocolomotor disorders in participants with PD, we resorted to calculating saccade probability and duration. An impairment in saccadic initiation, leading to a more variable onset of saccadic movements than matched healthy controls, has been shown to characterize patients PD from early on in the disease progression (Terao et al., 2011). Furthermore, saccade intrusions—characterized by involuntary saccades away and back to a fixation point, characterize oculomotor system functioning in PD (White et al., 1983), adding to variance in saccade probability distribution. We selected a probabilistic algorithm which detects saccades—as distinct from peri-blink saccadic movements—using an unsupervised training period (between 50 and 200 s), and uses expectation maximization to learn the parameters of Gaussian likelihood distributions for saccades (Toivanen et al., 2015; https://github.com/bwrc/eogert). Two parameters were selected: saccade probability for each detected event, and saccade duration. A Wilcoxon rank sum test was used to detect significant differences in mean variance between PD and HC.

## Results

###  Blink Models

**Figure 2:**
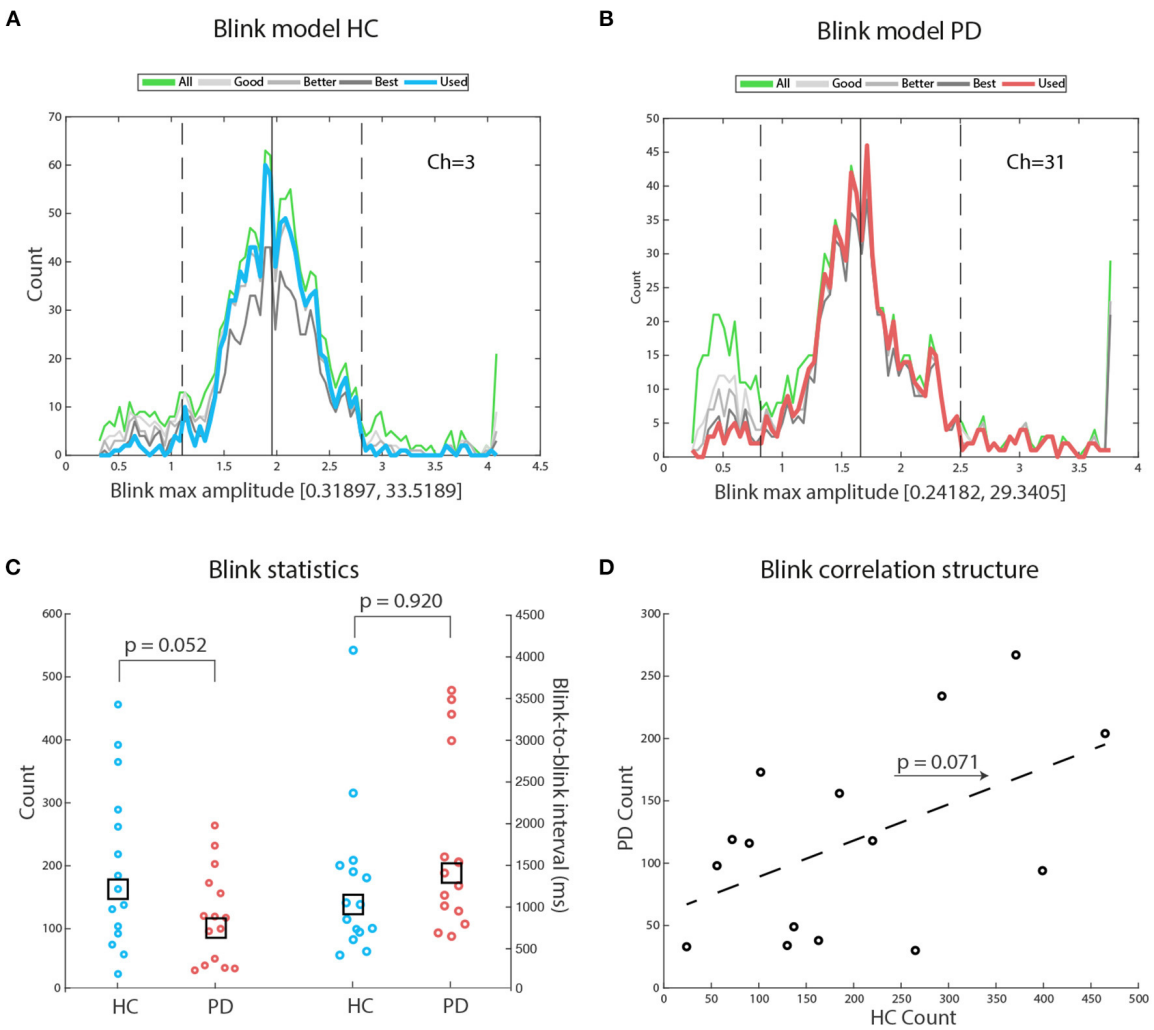
Blink models: **(A)** Exemplary blink models for participant number 1 of the HC group. The green line depicts the amplitude distribution of all potential blinks: Amplitudes are measured in standard deviations, to avoid the confounding effects of differences in mean blink amplitudes across participants. Blink range on the x-axis: Notice that the right tail of the distribution is interrupted because most large amplitude values were outliers. The blue line depicts the blink distribution selected for further analysis. For details, see the Materials and Method section. **(B)** Exemplary blink models for participant #1 of the PD group. The red line depicts the blink distribution selected for further analysis. **(C)** There was a tendency to a significant difference favoring HC in total blink counts. There was no significant difference between HC and PD on mean interblink interval. Notice that the median interval is similar across groups. The largest median blink interval for both groups corresponds to physiological intervals. **(D)** A robust regression fit shows a tendency for matched participants from both groups to perform similarly, hinting at possible underlying common factors driving blink frequency.

Figures 2A,B display illustrative blink modeling results for participant number 1 of both groups. In both cases, the right tail of the distribution contains outliers that are eliminated based on the distance from the best blinks distribution (up-stroke and down-stroke R2 > 0.98). A Wilcoxon rank sum test of the difference between the number of blinks in HC and in PD failed to reach significance: *Z* = 1.61, *p* = 0.052, HC median number of blinks = 163, PD median number of blinks = 116. We then checked for the physiological realness of the interblink intervals, and found that values for both groups were comparably within expected values: HC median interblink interval = 1,037 ms (range: 650–3,638), PD median interblink interval = 1,419 ms (range: 422–4,125), *Z* = –1.41, *p* = 0.920. With the exception of one participant in each group, all medians were below 3,500 ms, likely reflecting the chunking effect of attention to the repeating tone sequence (see Figure 2C). The concentration of individual median values at the lower portion of the range suggests an attractive effect of stimulus rate on blink rate. A robust regression fit between HC blink counts and PD blink counts failed to reach significance [*t*_(13)_ = 1.959, *p* = 0.071] (see Figure 2D). When we averaged blink counts across groups and regressed the results against age in years, we found no significant fit (Spearman ρ = –0.215, *p* = 0.503), suggesting that in our samples age did not appear to be driving changes in blink frequencies.

###  Blink Distribution Reflects Stimulus Structure

**Figure 3:**
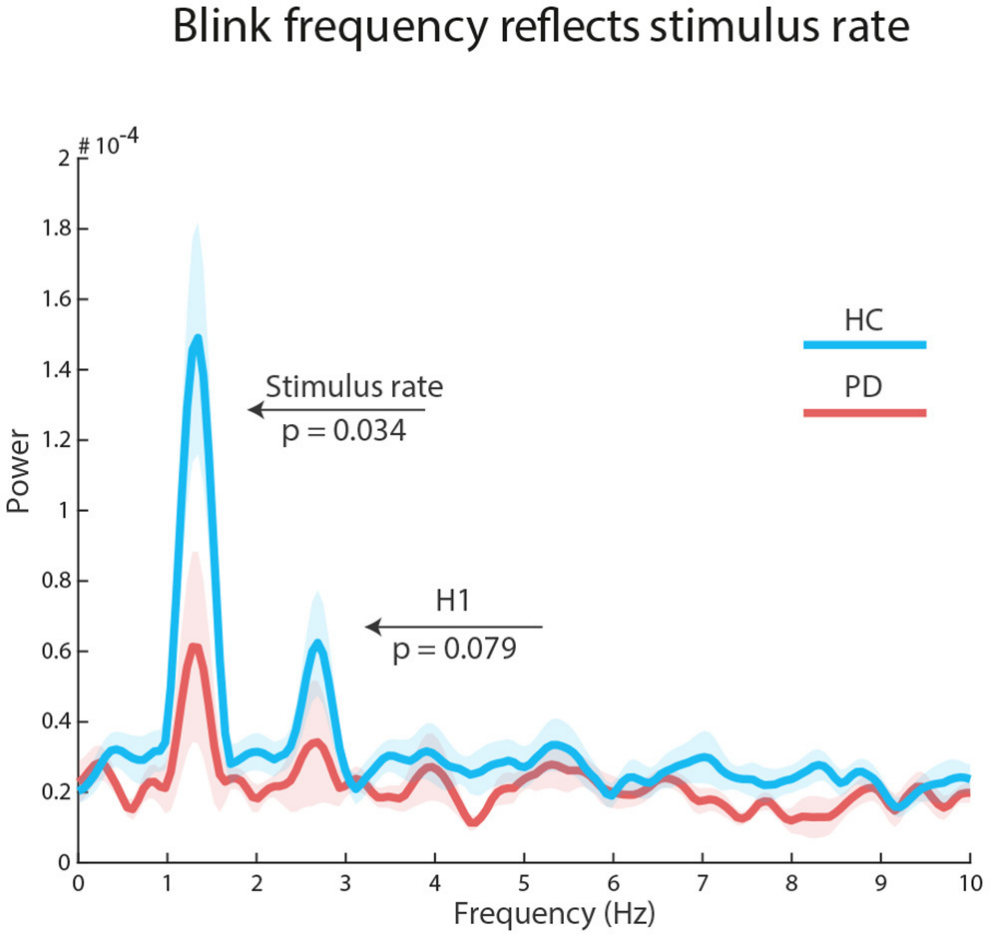
Auditory regularities in the eyes: FFT results on concatenated epochs show a significant effect of stimulus rate in both groups, but larger for healthy control participants (blue line) than participants with Parkinson's Disease (red line). The first harmonic processes (H1) were not significant.

To explore how stimulus structure influenced the temporal distributions of blinks, we concatenated all selected epochs and submitted the resulting vector to a Fast Fourier Transform (FFT) analysis. Using a Wilcoxon signed rank test, we found a significant peak at stimulation frequency (1.34 Hz) in each group: HC, *Z* = 2.89, *p* = 0.002, *r* = 0.74 (reference power = 3*10^-05^); PD, *Z* = 1.98, *p* = 0.047, *r* = 0.51 (reference power = 2*10^-05^). However, there was a significant difference in peak power between the groups: HC median = 1.098*10^-04^, PD median = 4.644*10^-05^, *Z* = 1.825, *p* = 0.034, *r* = 0.33. There was no significant group peak, nor a group difference at the first harmonic of the stimulus rate (2.67 Hz): all ps ≥ 0.079. Similarly, there were no significant findings at the repeating sequence frequency (0.266 Hz): all ps ≥ 0.187 (see Figure 3).

###  Blink Rates Encode Temporal Predictions

**Figure 4:**
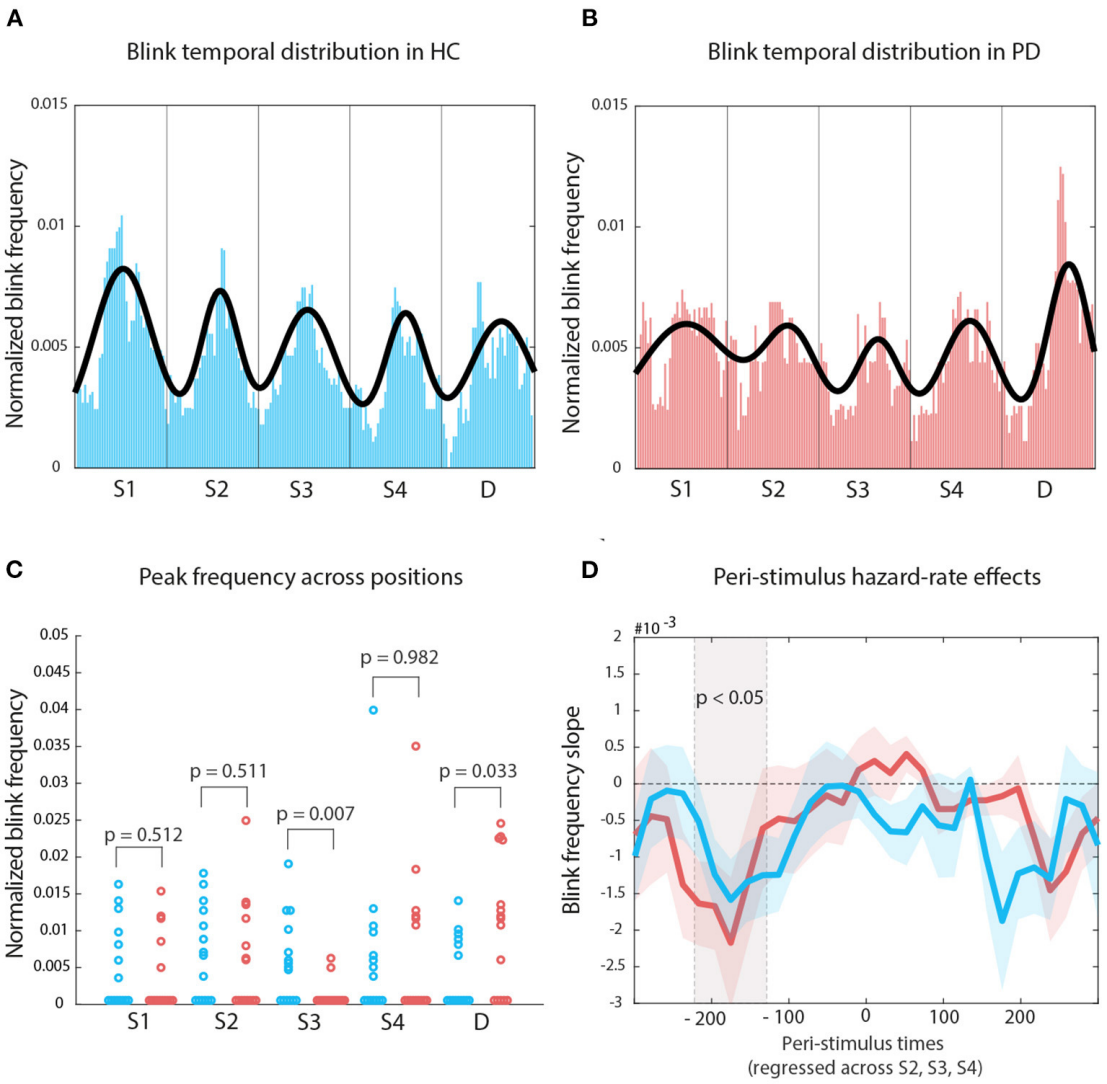
Temporal organization of blink onset: **(A)** Median of blink frequency medians estimates across time for HCs. The Gaussian fit highlights the regularity in blink peak distribution following entrainment to stimulus regularity. **(B)** The same approach for PD participants displays a less organized structure. **(C)** The main difference between the two distribution lies with how blinks reflect the onset of S3. **(D)** The gray shade indicates mean blink frequency slopes for both HC and PD which display significant oculomotor inhibition effects; colored shaded areas indicate standard error of the mean.

The Nelder-Mead algorithm allowed us to optimally fit 5 Gaussians on the median of the median blink distributions for each group. For HC, the number of iterations was 719, with a mean residual of 6.447*10^-4^. For PD, the number of iterations was 1228, with a mean residual of 7.515*10^-4^ (see Figures 4A,B, respectively). We measured histogram similarity using χ^2^ as a distance measure, and found that—globally—the distribution of blinks across the repeating sound sequence did not differ (distance = 1.494, *p* = 0.73, bootstrapping distribution, 1,000 repetitions). However, when we tested the differences in blink frequency (pristine values, that is before applying the moving-average smoothing) at fitted curve peak within each sound interval, using the fitted values for HC as reference also for participants with PD, we then found that the two groups differed in the S3 interval, that is at the center of the sequence (original *p* = 0.007, FDR *p* = 0.01), which corresponds also to the middle point in the attentive searchlight for a potential target onset (Figure 4C).

We then used a robust linear fit approach to calculate, for each peristimulus bin point, the slope of values across the onset of sounds at S2, S3, and S4. For both groups, we found a significant hazard-rate effect on blink frequency in the pre-stimulus period only (Figure 4D): all cluster Ts *leq* –2.79, all cluster ps *leq* 0.029; HC –220 to 200 ms, PD –180 ms; negative slopes indicate the amount of decrease with each potential Target interval. Pre-stimulus oculomotor activity became more inhibited with increasing waiting time across positions S2, S3, and S4.

###  Saccadic Movements

The similarity in blink temporal distribution between participants with PD and HCs becomes more relevant on the background of the significant difference between the two groups in saccadic movements estimated from electrooculographic data. Average saccade probability displyed larger variance in participants with PD (mean = 0.014) than in HCs (mean = 0.011): *Z* = 2.13, *p* = 0.032, *r* = 0.54. However, average saccade duration did not differ: *Z* = 0.43, *p* = 0.663. This suggests a disorder in saccade initiation in participants with PD, detectable even in the context of isochronous auditory stimulation driving entrainment in blink onset.

## Discussion

When stimulus statistics in the environment drive our attention toward the potential onset of a target event, changes occur at both central and autonomic nervous system levels, thereby modulating all motor effectors, not just those required to press a button. Indeed, recent work suggests that temporal predictions are reflected by eye movements, such as saccades and blinks (Abeles et al., 2020), that are partially under voluntary and partially under involuntary control. When we approach the probable onset time of a target event, ocular movements are suppressed, in order to avoid diverting attention to other stimuli (saccades) or suppressing sensory input (blinks). However, in everyday situations the uncertainty about when a target event will occur adds to the uncertainty about whether a target will occur at all. We tested whether oculomotor inhibition occurs for targets whose chance is globally very low (20%). Furthermore, by comparing the performance of healthy controls (HC) and gender- and age-matched Parkinson's Diseases (PD) patients, we measured the extent to which temporal predictions conveyed via oculomotor inhibition depend on general oculomotor fitness, which is impaired in PD. Overall, PD participants tended to produce less blinks than HCs, but the temporal organization of inter-blink-intervals is similar to that of healthy controls. Previous work showed that, in spontaneous blinking conditions and for a cohort between 40 and 89 years, mean blink amplitude and peak velocity decreased with age, but blink rate as such was not affected (Sun et al., 1997). In our case, age was not a significant factor in determining blink counts, although the effect of age on blink counts might have been overridden by the entraining effects of the stimulation structure.

We also found that inter-blink-intervals in both groups tend to follow the regular auditory stimulation rate (750 ms, 1.34 Hz, Figure 3), although HC outperformed participants with PD. This finding suggests that motor impairment in our sample of participants with PD, including oculomotor saccadic impairments, did not prevent the locking of blink frequency to stimulus statistics. However, oculomotor impairment in PD partially affected the organization of blink peak distribution (Figures 4A–C). Although the sequence-based distance between blink histograms for HC and PD was not significant, we found that in participants with PD blinks were significantly less likely to occur in response to the third sound of each repeating sequence. Previous work showed that spontaneous blinks in PD participants with mild and moderate severity were either abnormally reduced or increased relative to HC (Korosec et al., 2006). PD participants in that study displayed a more advanced motor impairment (UPDRS motor scale score) than in our patient sample, and participants were tested off medication, while the patients in our sample were tested on medication. The lack of an off medication condition is a limit to our findings, as it would have provided a test for blink entrainment. However, our study assesses oculomotor functionality within a continuous attention condition, that is under under stressful attentional demands (Maffei and Angrilli, 2018), suggesting resilience in patients' performance.

When we regressed blink probability across potential Target positions (S2, S3, S4), we found evidence of a hazard rate organization of blink onset probability in both groups. Oculomotor inhibition progressively increased while waiting for a potential target (Figure 4D). Importantly, as our analysis was run on the repeating sequences that did not contain a target, oculomotor inhibition was purely driven by cognitive expectancies for future target onset. The “hazard rate” of oculomotor activity is evident in the prestimulus period only, consistent with previous findings (Tavano et al., 2019; Abeles et al., 2020). Motor disorganization as a consequence of PD—at least in as far as it affects the oculomotor system—did not prevent the computation of evolving target probability in time, which is a key component of the processes generating temporal expectations. This likely preserves in patients a sufficient fit with the environment, whose statistics are inherently time-dependent.

## Data Availability Statement

The raw data supporting the conclusions of this article will be made available by the authors, without undue reservation.

## Ethics Statement

The studies involving human participants were reviewed and approved by Ethics Committee of the University of Leipzig, Germany. The patients/participants provided their written informed consent to participate in this study.

## Author Contributions

AT and SK devised research hypothesis, wrote and revised draft, and approved submission. AT created scripts for data collection and analyzed data. Both authors contributed to the article and approved the submitted version.

## Conflict of Interest

The authors declare that the research was conducted in the absence of any commercial or financial relationships that could be construed as a potential conflict of interest.

## Publisher's Note

All claims expressed in this article are solely those of the authors and do not necessarily represent those of their affiliated organizations, or those of the publisher, the editors and the reviewers. Any product that may be evaluated in this article, or claim that may be made by its manufacturer, is not guaranteed or endorsed by the publisher.
